# Imbalance between the caudate and putamen connectivity in obsessive–compulsive disorder

**DOI:** 10.1016/j.nicl.2022.103083

**Published:** 2022-06-14

**Authors:** Ziwen Peng, Tingxin He, Ping Ren, Lili Jin, Qiong Yang, Chuanyong Xu, Rongzhen Wen, Jierong Chen, Zhen Wei, Tom Verguts, Qi Chen

**Affiliations:** a Key Laboratory of Brain, Cognition and Education Sciences, Ministry of Education, China; b School of Psychology, Center for Studies of Psychological Application, and Guangdong Key Laboratory of Mental Health and Cognitive Science, South China Normal University, 510631 Guangzhou, China; c Department of Geriatric Psychiatry, Shenzhen Kangning Hospital, 518020 Shenzhen, China; d Southern Medical University, 510515 Guangzhou, China; e Affiliated Brain Hospital of Guangzhou Medical University, 510370 Guangzhou, China; f Department of Child Psychiatry and Rehabilitation, Affiliated Shenzhen Maternity & Child Healthcare Hospital, Southern Medical University, 518017 Shenzhen, China; g Department of Experimental Psychology, Ghent University, 9000 Ghent, Belgium

**Keywords:** Obsessive-compulsive disorder (OCD), Functional connectivity, Caudate connectivity, Putamen connectivity

## Abstract

**Background:**

Compulsive behaviors in obsessive–compulsive disorder (OCD) have been suggested to result from an imbalance in cortico-striatal connectivity. However, the nature of this impairment, the relative involvement of different striatal areas, their imbalance in genetically related but unimpaired individuals, and their relationship with cognitive dysfunction in OCD patients, remain unknown.

**Methods:**

In the current study, striatal (i.e., caudate and putamen) whole-brain connectivity was computed in a sample of OCD patients (OCD, n = 62), unaffected first-degree relatives (UFDR, n = 53) and healthy controls (HC, n = 73) by ROI-based resting-state functional magnetic resonance imaging (rs-fMRI). A behavioral task switch paradigm outside of the scanner was also performed to measure cognitive flexibility in OCD patients.

**Results:**

There were significantly increased strengths (Z-transformed Pearson correlation coefficient) in caudate connectivity in OCD patients. A significant correlation between the two types of connectivity strengths in the relevant regions was observed only in the OCD patient group. Furthermore, the caudate connectivity of patients was negatively associated with their task-switch performance.

**Conclusions:**

The imbalance between the caudate and putamen connectivity, arising from the abnormal increase of caudate activity, may serve as a clinical characteristic for obsessive–compulsive disorder.

## Highlights

• The imbalance between the caudate and putamen connectivity in OCD patient arises from the abnormal connection of caudate.
• The abnormal caudate connectivity mainly results from the outward extension of cortico-striato-thalamo-cortical loop.
• The caudate connectivity of OCD patients is negatively associated with their task-switch performance.

## 1 Introduction

Obsessive-compulsive disorder (OCD) is surprisingly common in the general population (2.5%–3%) with a high genetic risk (Robbins et al., 2019). This disorder is characterized by persistent obsessions and compulsions. Specifically, compulsion (persistent behavior, despite negative consequences) is one of the core manifestations of the disorder, and plays an important role in the mechanism of OCD.

For understanding the pathomechanism of compulsive behaviors, we need to know its cognitive and neural foundations (Figee et al., 2016, Gillan et al., 2016, Gillan and Robbins, 2014, Robbins et al., 2019, Simmler and Ozawa, 2019), in particular the cortico-striatal circuits (Dong et al., 2020, Heilbronner et al., 2016, Morelli et al., 2011, Nagarajan et al., 2018, Pinhal et al., 2018). Previous studies proposed that the connectivity from cortex to dorsomedial (caudate) and dorsolateral striatum (putamen) constitute parallel loops, namely the associative and motor loops, respectively (Middleton, 2000, Milad and Rauch, 2012, O’Doherty et al., 2017, Seger, 2018, Yin and Knowlton, 2006). The associative loop connecting the caudate and prefrontal cortex is associated with goal-directed and flexible behaviors (Dong et al., 2020, Seger, 2018, Simmler and Ozawa, 2019). The motor loop connecting putamen with premotor and sensorimotor cortex is vital in habits (de Wit et al., 2012, Seger, 2018). Since compulsivity has been characterized as an imbalance between the brain's goal-directed and habit-learning systems (Gillan et al., 2016), these loops are thought to mediate cognitive control of learning and behavior, and its automatization into habits. It is understandable then that abnormal or excessive active avoidance has been perceived as a key impairment in OCD (Geramita et al., 2020).

Converging evidence indicates that compulsive behaviors may be associated with an imbalance between the associative and motor loops (Dolan and Dayan, 2013, Dong et al., 2020, Gillan et al., 2014b, Gillan and Robbins, 2014). When functioning properly, these two systems shift flexibly according to the external environment and feedback. Disruption of either of the two loops could result in an imbalance between them, and consequently the emergence of compulsive behavior (Banca et al., 2015, Dong et al., 2020, Gillan et al., 2014a, Gillan et al., 2014b, Gillan et al., 2011). However, it’s unclear which of the two loops (or both) drives the imbalance. Few studies have investigated the direct relationship between these two systems; however, such study could promote our understanding of their interaction in OCD (Geramita et al., 2020).

The risk of OCD is significantly higher for relatives of patients (Gottesman and Gould, 2003, Nestadt et al., 2010, Pauls, 2008, Pauls et al., 2014). There could thus be a genetic basis to OCD, although the specific gene and its relation with behavior remains elusive. The concept of endophenotype (Gottesman and Shields, 1973) was adopted to understand the gap between genetics and behavioral disease processes. Behaviorally, OCD patient probands and their unaffected first-degree relatives (UFDR) showed cognitive inflexibility (Chamberlain et al., 2007). Neurally, OCD patients and their UFDR exhibit an associated reduction or increase in brain functional and structural variation involved in the associative loop, compared to healthy controls (HC)(Menzies et al., 2007). Specifically, patients and their UFDR show leftward asymmetry of cortical thickness in the anterior cingulate cortex (ACC) (Peng et al., 2015). Another investigation identified reduced activation of orbitofrontal cortex, during reversal learning in patients and their UFDR (Chamberlain et al., 2008). A recent resting-state fMRI (rs-fMRI) study of our lab found that patients and UFDR showed greater effective connectivity between the left caudate and frontal cortex than HC (Dong et al., 2020). These results indicate that the abnormal activity or connectivity in the associative loop may be a hereditary risk factor for OCD. Considering that cognitive flexibility is an identified endophenotype of OCD (Robbins et al., 2019), it’s critical to clarify this loop as a whole and its relation with cognitive flexibility.

Currently, based on the previous study where we investigated the effective connectivity of specific areas only, we used rs-fMRI to measure whole-brain functional connectivity of caudate and putamen in OCD, UFDR and HC groups. First, we investigated the imbalance between caudate and putamen connectivity in OCD and measured whether it was also observed in UFDR of OCD patients, which could thus serve as a clinical characteristic for this disorder. Second, we explored whether the severity of obsessive–compulsive symptoms could influence the interaction between the two (caudate and putamen) systems. Finally, previous studies reported that goal-directed associative learning requires people to flexibly use feedback according to task demands. Cognitive inflexibility might prevent patients from shifting from one thought to another, thus rendering their behavior stimulus-driven and inflexible (Mayr and Keele, 2000, Monsell, 2003). Thus, we investigated whether cognitive inflexibility is related to these two loops.

## 2 Materials and Methods

### 2.1 Participants

Healthy controls (n = 73; males = 51), OCD patients (n = 62; males = 45) and their unaffected first-degree relatives (n = 53; males = 32) were recruited for the study from Guangzhou Psychiatric Hospital. All participants, aged 18 to 55, underwent the diagnosis performed by a clinical psychiatrist and an experienced psychologist. In accordance with the institutional research and ethics committee of Guangzhou Psychiatric Hospital, each subject gave written informed consent after understanding the complete study description. Both OCD patients and their UFDR were recruited in the hospital. All procedures contributing to this work comply with the ethical standards of the relevant national and institutional committees on human experimentation and with the Helsinki Declaration of 1975, as revised in 2008.

The patients with OCD received the Structured Clinical Interview (SCID) for DSM-IV-TR Axis I disorders (First et al., 2002), fulfilling Diagnostic and Statistical Manual of Mental Disorders, 4th edition (DSM-IV) criteria for OCD. Their unaffected first-degree relatives and healthy controls were assessed by the SCID for the DSM-IV-TR Axis I disorders, Research Version, Non-Patient edition (SCID-I/NP) (Spitzer et al., 2002). All participants had no history of traumatic brain injury or neurological disease and did not exhibit alcohol/substance abuse. The UFDR of patients and healthy controls were excluded if they reported any history of mental illness and/or treatment with any psychotropic medication. On this basis, healthy controls had an additional exclusion criterion of no family history of Axis I or Axis II psychiatric disorders.

Thirty-one OCD patients took normal psychotropic medications while scanning (see Supplementary Material, Table S1). Twenty patients had comorbid disorders such as anxiety or depression. It is worth noting that having only comorbid anxious and depressive symptoms were not considered as an exclusion criterion, provided that OCD was the primary clinical diagnosis.

### 2.2 Clinical assessments

The Yale-Brown Obsessive-Compulsive Scale (Y-BOCS) (Goodman et al., 1989) was administered to assess illness severity. The Obsessive-Compulsive Inventory-Revised (OCI-R) (Foa et al., 2002, Peng et al., 2011) was used for measuring the degree of distress or being disturbed with common OCD phenomena (Fernandez-Egea et al., 2018). The Beck Depression Inventory (BDI) (Beck and Steer, 1984) was used to estimate depressive symptoms, and the State-Trait Anxiety Inventory (STAI)(Spielberger, 1989) was used to measure anxiety symptoms. Individual clinical assessment measures were compared between or across groups using chi-square analyses and Analysis of Variance in SPSS version 20.0.

### 2.3 Imaging data acquisition

All MRI data were acquired on a 3.0-Tesla MR system (Philips Medical Systems Nederland B.V.) equipped with an eight-channel phased-array head coil. The resting-state functional MRI data were collected with the following parameters: gradient echo Echo-Planar Imaging (EPI) sequences; time repetition, TR = 2000 ms; echo time, TE = 30 ms; flip angle = 90°, 33 slices, field of view [FOV] = 220 mm × 220 mm, matrix = 64 × 64; slice thickness = 4.0 mm; voxel size = 3.4 × 3.4 × 4 mm^3^. For each participant, the fMRI scanning lasted for 480 s and generated 240 whole-brain volumes. During the scanning, participants were instructed to lie quietly with their eyes closed, and stay awake without moving. For spatial normalization and localization, the high-resolution T1-weighted anatomical images were obtained by using a magnetization prepared gradient echo sequence with the following parameters: TR = 8 ms, TE = 3.7 ms, flip angle = 7°, FOV = 240 mm × 240 mm, matrix = 256 × 256, slice thickness = 1.0 mm; voxel size = 1.0 × 1.0 × 1.0 mm^3^.

#### 2.3.1 Functional imaging data preprocessing

The data was preprocessed using the Statistical Parametric Mapping toolbox (SPM12, https://www.fil.ion.ucl.ac.uk/spm), and Data Processing Assistant for Resting-State fMRI (DPARSFA version 4.4, https://rfmri.org/dpabi). For image preprocessing, the first 10 time points were removed to denoise the signal. The remaining 230 volumes were corrected for slice timing and head motion, as all the subjects had no>1.5° of maximal rotation and 1.5 mm of maximal translation. After realignment with the corresponding T1-volume and visual inspection as the image quality control method to exclude non-conforming images, the nuisance covariates (six head motion parameters, white matter signal and cerebrospinal fluid signal) were regressed in first-level analysis. Next, the functional data were normalized into the stereotactic space of the Montreal Neurological Institute and resampled at 3 × 3 × 3 mm^3^. The processed images were spatially smoothed with a 6-mm full-width half-maximum isotropic Gaussian kernel. Further preprocessing pipeline consisted of band-pass filtering (0.01–0.08 Hz) to reduce the effects of physiologic noise and micro-head-motion correction according to frame-wise displacement (FD) by replacing the rs-fMRI volume with FD > 0.5 mm (nearest neighbor interpolation).

#### 2.3.2 Caudate connectivity and putamen connectivity construction

Two ROIs for functional connectivity were defined according to previous studies (Di Martino et al., 2008, Dong et al., 2020). The caudate ROI was constructed using the MNI-coordinates: X = ±13, Y = 15, Z = 9; the MNI-coordinates of the putamen ROI were: X = ±28, Y = 1, Z = 3. These ROIs were defined by spheres surrounding the central voxel with a radius of 3 mm. A previous study includes additional details about the anatomical delineation of these regions (Di Martino et al., 2008). We drew a plot to show the location of two spherical ROIs to demonstrate there is no overlap between them (see Supplementary Material, Fig. S3). All anatomical regions mentioned in the results of the study are identified by the automated anatomical labeling (AAL) atlas.

The averaged time course within each seed was extracted and correlated with all voxels in the entire brain to generate functional connectivity for the putamen and the caudate. Due to anatomical proximity, the caudate was regressed out as a covariate when analyzing the putamen, and vice versa. Then the Pearson correlation coefficients (r) between ROI activation and each voxel were subsequently Fisher-Z transformed: Z = 0.5 × ln((1 + r)/(1-r)). One-sample *t* tests in each group on functional connectivity maps for the caudate connectivity and putamen connectivity were conducted respectively, using the rest toolbox (https://restfmri.net/forum/REST_V1.8). Then, we obtained six connectivity maps as masks by False Discovery Rate used for cluster-level multiple comparisons correction (*p* < 0.001, FDR corrected). Afterwards, a union mask of the caudate connectivity including all significant connections in any of the three groups was produced, and another union mask of putamen connectivity was generated in the same way. Then each subject obtained the final connectivity maps of the two brain regions by multiplying the data of their two raw functional connectivity maps with the corresponding union mask respectively. Finally, the functional connectivity values (Z) were averaged for supra-threshold caudate and putamen connectivity separately. We referred to the mean Fisher-Z transformed value in caudate connectivity map as caudate connectivity strength; and the mean Fisher-Z transformed value in putamen connectivity map as putamen connectivity strength. Hence, each of the two ROIs (caudate, putamen) for each group, has a connectivity map (FDR corrected) and a mean functional connectivity strength value for further analysis that employed Analysis of Variance (ANOVA) and two-sample post hoc *t* tests to analyze the differences between the three groups. Gaussian Random Field theory (GRF) was used for cluster-level multiple comparisons correction (cluster level *p <* 0.05, voxel level *p <* 0.001, corrected).

### 2.4 Task-switching paradigm

The task-switching paradigm was used to investigate cognitive flexibility (Gu et al., 2007). This paradigm was conducted outside the fMRI scanner (see Supplementary Material, Fig. S2), in the OCD patients. Participants were required to learn associations between stimuli and responses. A total of 264 trials were presented. Half of the trials were task-switching conditions, during which the subjects attended to a different dimension as on the previous trial (e.g., a square cue followed by a diamond cue); the other half of the trials were task-repeat conditions during which the dimensions were the same as on the previous trial. During the 10-minute training phase, participants were required to practice and learn the stimulus-action mapping. During the test phase, reaction time (RT) and accuracy were recorded. Switching cost (task-switch RT minus task-repeat RT) was used as a measure of cognitive flexibility, which meant the lower an individual’s switching cost, the better his cognitive flexibility. Only the correct responses are included in the following analyses. The experimental flow was the same as in a previous study which explored abnormal brain activity related to cognitive inflexibility in OCD (Gu et al., 2007).

### 2.5 Brain-behavior correlation analysis

Moderation analyses were performed between mean caudate connectivity, mean putamen connectivity, and Yale-Brown Obsessive-Compulsive Scale (YBOCS) scores to examine associations between functional connectivity and OCD symptom severity. All variables are zero-centered.

Correlation analyses were performed between mean strength of the ROI-based connectivity and task-switching performance in the OCD group, to examine associations between functional connectivity and cognitive flexibility (RT and accuracy data exceeding plus or minus three standard deviations are excluded). The moderation and correlation analyses were conducted using SPSS 20.0.

## 3 Results

### 3.1 Demographic and clinical analysis

The demographic and clinical variables of each participant group are presented in Table 1. There were no differences in age (F(2,185) = 2.4, *p* = 0.10), gender (χ^2^ = 2.1, *p* = 0.34) or education level (F(2,185) = 0.1, *p* = 0.87) across the three groups. The head motion, the YBOCS, the OCI-R, the BDI, and the STAI of OCD patients were significantly higher compared to the HC group and the UFDR group. Since age, gender, education, head motion, BDI and STAI may play roles in differences between groups, they were treated as nuisance variables and controlled in all subsequent data analysis.

**Table 1:** Demographic and clinical variables of participants.

Measures	OCD (n = 62)	UFDR (n = 53)	HC (n = 73)	F/χ^2^	*p*
Demographic Measures					
Age (years) (SD)	26.8(8.3)	23.9(8.3)	27.2(9.4)	2.4	0.10
Gender (Male: Female)	45:17	32:21	51:22	2.1	0.34
Education (years) (SD)	12.77(3.5)	11.4(3.1)	11.6(3.3)	0.1	0.87
Head motion (SD)	0.0673 (0.03954)	0.0574 (0.02799)	0.0517 (0.02756)	4.0	<0.05

Clinical Measures					
YBOCS total (SD)	29.0(4.9)	3.8(3.3)	0(0)	32.9	<0.001
YBOCS obsessions (SD)	14.6(2.9)	1.8(2.0)	0(0)	27.8	<0.001
YBOCS compulsions (SD)	14.4(3.1)	1.9(2.1)	0(0)	24.7	<0.01
OCI-R (SD)	21.8(13.6)	14.5(13.1)	14.8(13.3)	5.9	<0.01
BDI(SD)	16.4(12.6)	7.9(5.7)	5.9(5.5)	27.3	<0.001
STAI state (SD)	49.0(20.7)	29.5(15.2)	25.8(17.6)	30.2	<0.001
STAI trait (SD)	50.0(20.4)	39.2(19.6)	33.6(15.4)	13.6	<0.001

OCD: Obsessive–compulsive disorder group; UFDR: unaffected first-degree relative group; HC: healthy control group. YBOCS: Yale-Brown Obsessive-Compulsive Scale; OCI-R: Obsessive Compulsive Inventory–Revised; BDI: Beck Depression Inventory; STAI: State-Trait Anxiety Inventory. SD: standard deviation.

### 3.2 Caudate connectivity and putamen connectivity

**Fig. 1:**
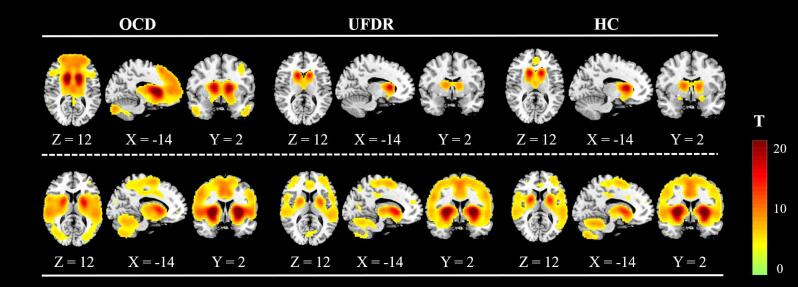
Caudate and putamen connectivity maps in three groups. Whole-brain connectivity patterns. Upper row: Connectivity with the caudate. Lower row: Connectivity with the putamen. The activation is mapped on the MNI template. Color bars represent the T-value (FDR correction, *p <* 0.001, corrected).

We measured the caudate and putamen whole-brain connectivity patterns in all participants by one-sample *t* tests, and found the caudate connectivity map for all three groups included the ventral medial prefrontal cortex (vmPFC), the orbitofrontal cortex (OFC), the ACC, the dorsolateral prefrontal cortex (DLPFC) and an area of the cerebellum (Fig. 1, upper row). The putamen connectivity map for all three groups included the supplementary motor area (SMA), the insular cortex, the primary motor cortex and an area of the cerebellum (Fig. 1, lower row). Based on visual inspection (i.e., magnitude and distribution of T-values), the pattern of UFDR group was more similar to that of HC group for both caudate and putamen connectivity.

**Fig. 2:**
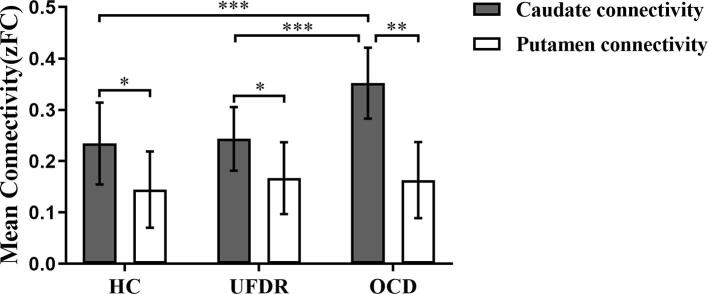
The mean caudate and putamen connectivity strength in three groups. Fisher-Z transformed mean connectivity strength for the two ROIs in the three groups. Error bars represent standard error of the mean (SEM). * *p <* 0.05; **p < 0.01; *** *p <* 0.001.

Then, we conducted a mixed design two-factor repeated measures ANOVA analysis to investigate the differences in caudate and putamen connectivity strength among OCD, UFDR and HC groups. The two factors in this model are group (OCD, UFDR, and HC) and network (or map) type (caudate, putamen), and the dependent variable is the mean connectivity strength values in these two maps. We observed a significant group main effect, F(2,104) = 22.357, *p* < 0.001, *η^2^* = 0.200; and a network type main effect, F(1,52) = 9.748, *p* < 0.001, *η^2^* = 0.052. The group-network type interaction effect is also significant, F(2, 104) = 19.800, *p* < 0.001, *η^2^* = 0.181. Specifically, for putamen connectivity strength, there was no difference between any of the groups (F (2,104) = 1.573, *p =* 0.210, *η^2^* = 0.017); but for caudate connectivity strength, there was a significant difference between groups (F (2,104) = 41.808, *p* < 0.001, *η^2^* = 0.318) (Fig. 2).

### 3.3 Moderation analysis

#### 3.3.1 Group differences in correlation between caudate and putamen connectivity

**Fig. 3:**
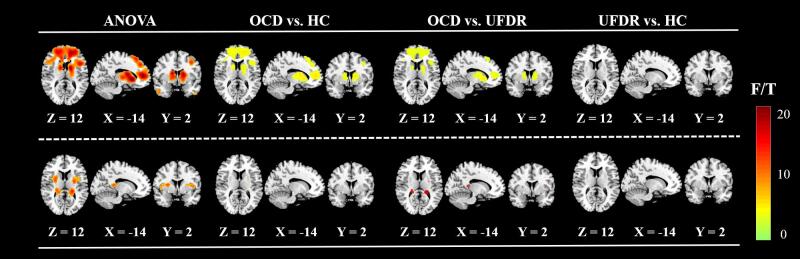
Group differences in correlation between caudate and putamen connectivity. Group comparison based on caudate connectivity and putamen connectivity. Upper row: Group difference in the caudate connectivity. Lower row: Group difference in the putamen connectivity. The activation is mapped on the MNI template. ANOVA, Analysis of Variance. Color bars represent both the F-value of the ANOVA and the T-value of the post hoc two-sample *t* test (GRF correction, cluster level *p <* 0.05, voxel level *p <* 0.001, corrected).

We conducted ANOVA and subsequent t-tests based on the supra-threshold caudate and putamen functional connectivity maps between the three groups or between each pair of groups using the rest toolbox (Fig. 3). Combining the results of these two analyses (i.e., magnitude and distribution of F-values and T-values), the inter-group differences of the caudate connectivity maps in three groups were mainly contributed by the differences between the OCD group and the other two groups. The ANOVA results showed abnormal connectivities between caudate and several areas including the bilateral ACC, superior and inferior frontal gyri, inferior parietal gyri, inferior and middle temporal gyri, angular gyri and left inferior cerebellum (Fig. 3, upper row). In combination with the connectivity strength results mentioned above, the OCD group showed similar areas of abnormal activation compared to either the UFDR group or the HC group. The abnormal connectivities between the putamen and several areas were mainly concentrated in the bilateral insular cortex and hippocampus (Fig. 3, lower row).

**Fig. 4:**
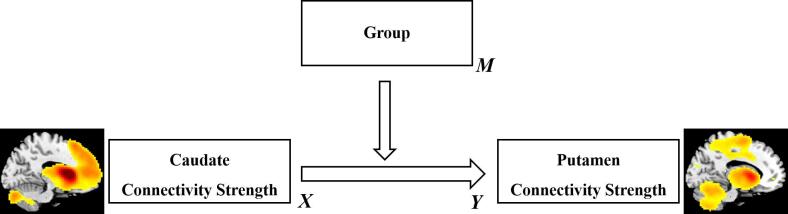
The moderation model of the group on the relationship between caudate and putamen connectivity.

In order to clarify whether the group modulated the correlation between caudate and putamen connectivity maps, a moderation analysis was performed to explore the influence of the group on the relationship between caudate and putamen connectivity strengths in the altered areas. The latter were defined as the regions obtained from the ANOVA between the three groups separately. The caudate connectivity strength significantly interacted with the group in predicting connectivity strength of the putamen in the OCD group (see Fig. 4., R2 = 0.075, F (1, 179) = 24.1462, *p* < 0.001). Since the moderator was a categorical variable, linear regressions of the independent variable (caudate strengths) on the dependent variable (putamen strengths) were further carried out separately for the three groups. It was found that the regression coefficients were significantly positive solely in the OCD group (OCD: R^2^ = 0.117, β = 0.342, *p* < 0.01; UFDR: R^2^ = 0.006, β = 0.080, *p* > 0.05; HC: R^2^ < 0.001, β = 0.012, *p* > 0.05). In sum, the significant correlation between the two types of connectivity strengths in the altered regions was observed only in the patient group, which may reflect the imbalance between the two networks.

### 3.4 Task switching analysis

Here we acquired behavioral results of 62 OCD patients. The error rate and mean reaction time (RT) under task repeat condition and task switch condition appear in Supplementary Material (Table S2). Both the repeat error rate and the switch error rate were significantly correlated with the Y-BOCS scores (r = 0.295, *p* = 0.030; r = 0.298, *p* = 0.029). Switching cost, reflecting cognitive flexibility, was calculated as mentioned before (task-switch RT minus task-repeat RT).

#### 3.4.1 Relationship between ROI-based connectivity and cognitive flexibility

**Fig. 5:**
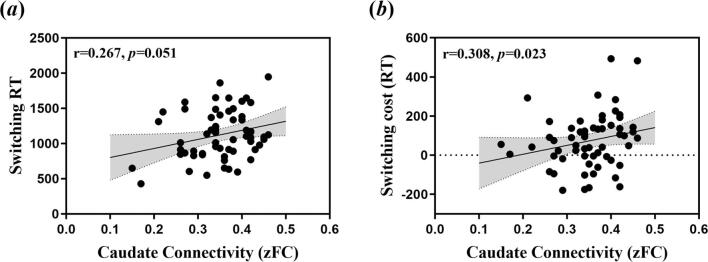
Correlations between mean connectivity strength and cognitive flexibility in the OCD sample. (a)The mean zFC (Fisher-Z transformed functional connectivity) value of the caudate connectivity in the OCD group was significantly positively correlated to switching cost (r = 0.267, *p =* 0.051). (b) The mean zFC (Fisher-Z transformed functional connectivity) value of the caudate connectivity in the OCD group was marginally positively correlated to switching RT (r = 0.308, *p =* 0.02). The gray area represents the interval of 95% confidence.

We investigated the relationship between functional connectivity strength of whole brain and cognitive flexibility. Longer task-switch RT was significantly associated with increased caudate connectivity strength, while the correlation between switching cost and caudate connectivity strength was marginally significant (see Fig. 5a and b; r = 0.308, *p =* 0.023; r = 0.267, *p* = 0.051). There was no significant correlation between putamen connectivity strength and switching cost (r = 0.062, *p =* 0.658). Since only OCD patients participated in the behavioral experiment, we were not able to assess these relationships between connectivity strength and cognitive flexibility in the UFDR group and HC group.

## 4 Discussion

The present study investigated the balance between the caudate and putamen connectivity in HC, OCD patients and their UFDR using rs-fMRI. Compared with HC and UFDR, OCD patients showed significantly increased caudate connectivity strength of the whole brain, but not putamen connectivity strength. These deficits represent trait rather than state impairments, that can exist in the absence of medication confounds and clinical phenotype (Chamberlain et al., 2007).The moderation analysis and linear regression for all three groups found a correlation between the two types of connectivity in OCD patients only, which may indicate that these two types of networks were imbalanced in this sense. In addition, there was a significant relationship between the caudate connectivity of patients and their RT in task-switch trials, suggesting that the abnormally activated caudate connectivity in OCD patients is associated with worse performance in the behavioral task.

Given the importance of caudate and putamen in goal-directed and habitual learning respectively (Balleine et al., 2007, Burton et al., 2015, Cabeza, 2002, Cox and Witten, 2019, Cushman and Morris, 2015, Di Martino et al., 2008, Dolan and Dayan, 2013, Dong et al., 2020), the abnormal correlations between two types of connections found in the OCD patients may support the hypothesis that compulsive symptoms are associated with an imbalance between the goal-directed and the habitual learning system. An increasing number of studies have found an imbalance between these two systems in OCD patients (Robbins et al., 2019, Simmler and Ozawa, 2019). Using a “slips-of-action” test, researchers found that the ability for understanding action outcome value changes was impaired in OCD patients compared to HC (Gillan et al., 2011). Furthermore, using a novel shock avoidance task designed to induce habits through goal-devaluation by overtraining, they observed that OCD patients have a tendency to develop excessive avoidance habits (Gillan et al., 2014b). Using fMRI, the authors further found that these increased avoidance habits in OCD patients were associated with hyper-activation in the caudate nucleus, a key region in the pathophysiology of OCD and goal-directed system (Gillan et al., 2015). These data suggest that habit-forming biases in OCD patients may arise from impairments in the goal-directed system. Together, these findings provide evidence that compulsion is associated with an imbalance between the goal-directed and habit-based system, caused by an impairment of the former system (Gillan et al., 2015).

Furthermore, the caudate and putamen connectivity maps were compared between OCD, UFDR and HC groups to investigate the neural differences. The frontal brain regions (i.e., bilateral ACC, superior and inferior frontal gyri), connected with the caudate showed significant group differences, belong to the cortico-striato-thalamo-cortical (CSTC) brain networks, specifically the frontal-striatal loop. Previous studies have found abnormal activation in this loop in OCD patients with difficulties in goal-directed behaviors (Dolan and Dayan, 2013, Howard and Kahnt, 2017, Medaglia et al., 2018, van der Plasse et al., 2007, Viviani, 2014). Although OCD is thought to be associated with a disruption of CSTC brain networks, some rs-fMRI studies have reported more diffuse alterations in brain connectivity in more extended resting state networks, extending from the inside of the CSTC loop to its outside (del Casale et al., 2011, Rotge et al., 2008, Thorsen et al., 2015). Recently, a systematic review and *meta*-analysis found that OCD patients showed increased emotional processing-related activation in limbic, frontal, and temporal regions (middle temporal cortices) (Thorsen et al., 2018). The increased connectivity between the caudate and temporal gyri (inferior and middle) may contribute to understand the complexity and diversity of cognitive and emotional deficits in OCD (Gonçalves et al., 2016). In addition, the role of the cerebellum in the pathophysiology of OCD has drawn increasing attention, both structurally (Brooks et al., 2016, Hu et al., 2017) and functionally (Sha et al., 2020, Xu et al., 2018). Consistent with the results in the present study, Moody’ s team have examined connectivity changes from rs-fMRI data before and after cognitive-behavioral therapy in OCD participants and healthy controls and found strong increases involving connectivity between the cerebellum and caudate/putamen (Moody et al., 2017). They suggested that this altered connectivity involving cerebellar to striatal and prefrontal regions may reflect acquisition of new non-compulsive goal-directed behaviors and thought patterns.

The caudate connectivity strength in the current study is correlated with performance in the task-switch trials. Combined with the results mentioned above, the increased caudate connectivity may mainly originate from central CSTC structures (caudate) to the regions outside the CSTC loop. In accordance with recent evidence, increased connectivity between the caudate and regions outside the CSTC (e.g., the parieto-occipital cortex, the angular gyrus and dorsolateral prefrontal cortex) may counterbalance the disconnectivity of the traditional CSTC loops in OCD (Fajnerova et al., 2020). Furthermore, the increased connectivity reported outside the CSTC (e.g., the frontal gyrus and temporal gyrus) could be related to visuo-spatial and sensory-motor processing, which may indirectly impact cognitive flexibility in OCD (Wolff et al., 2017). Our findings of a positive correlation between Y-BOCS and error rates during both repeat and switch events may seem surprising, but were consistent with previous studies reporting that differences in adaptation during repeat trials also played an important role (Han et al., 2011, Page et al., 2009, Woolley et al., 2008). One possible explanation is that the severity of OCD may be beneficial to accuracy at the expense of prolonged responding during repetition (Remijnse et al., 2013).

There are several limitations to the current study. First, the demographics of participants were not completely matched among the three groups. The sample was young and predominantly male, indicating an imbalance in gender distribution. Intelligence level, drug use, disease duration, co-morbidity and distinct subtypes of OCD patients may serve as confounding factors. Future research should better control these factors and/or examine the influence of these factors on OCD. Second, because of the limited manpower, the switch task was not administered in healthy controls. Third, the length of resting-state scanning could have been longer. The reliability of functional connectivity data may be fairly limited in the range of 5–10 min, suggesting the length of the resting state scan should be extended appropriately to obtain stable results. Lastly, this is a cross-sectional study. A longitudinal study would be important to obtain data related to the trajectory of network changes in OCD patients.

## 5 Conclusions

We identified the abnormally increased caudate connectivity in OCD patients mainly resulted from the outward extension of CSTC regions, which may be a compensatory mechanism for impaired CSTC loop in the disorder. The correlation between caudate and putamen connectivity was observed significantly only in OCD patients. These findings suggest the imbalance between the goal-directed and habitual cortico-striatal connectivity may serve as a clinical characteristic for OCD.

### CRediT authorship contribution statement

**Ziwen Peng:** Conceptualization, Methodology, Writing – original draft. **Tingxin He:** Data curation, Formal analysis, Writing – original draft. **Ping Ren:** Investigation, Writing – review & editing. **Lili Jin:** Data curation, Software, Writing – original draft. **Qiong Yang:** Project administration. **Chuanyong Xu:** Data curation, Visualization. **Rongzhen Wen:** Investigation. **Jierong Chen:** Investigation. **Zhen Wei:** Validation, Resources, Investigation. **Tom Verguts:** Writing – review & editing. **Qi Chen:** Supervision, Writing – review & editing.

## Declaration of Competing Interest

The authors declare that they have no known competing financial interests or personal relationships that could have appeared to influence the work reported in this paper.

## References

[b0005] Balleine B.W., Delgado M.R., Hikosaka O. (2007). The role of the dorsal striatum in reward and decision-making. J. Neurosci..

[b0010] Banca P., Voon V., Vestergaard M.D., Philipiak G., Almeida I., Pocinho F., Relvas J., Castelo-Branco M. (2015). Imbalance in habitual versus goal directed neural systems during symptom provocation in obsessive-compulsive disorder. Brain.

[b0015] Beck A.T., Steer R.A. (1984). Internal consistencies of the original and revised beck depression inventory. J. Clin. Psychol..

[b0020] Brooks S.J., Naidoo V., Roos A., Fouché J.-P., Lochner C., Stein D.J. (2016). Early-life adversity and orbitofrontal and cerebellar volumes in adults with obsessive-compulsive disorder: voxel-based morphometry study. Br. J. Psychiatry.

[b0025] Burton A.C., Nakamura K., Roesch M.R. (2015). From ventral-medial to dorsal-lateral striatum: neural correlates of reward-guided decision-making. Neurobiol. Learn. Mem..

[b0030] Cabeza R. (2002). Hemispheric asymmetry reduction in older adults: The HAROLD model. Psychol. Aging.

[b0035] Chamberlain S.R., Fineberg N.A., Menzies L.A., Blackwell A.D., Bullmore E.T., Robbins T.W., Sahakian B.J. (2007). Impaired cognitive flexibility and motor inhibition in unaffected first-degree relatives of patients with obsessive-compulsive disorder. Am. J. Psychiatry.

[b0040] Chamberlain S.R., Menzies L., Hampshire A., Suckling J., Fineberg N.A., del Campo N., Aitken M., Craig K., Owen A.M., Bullmore E.T., Robbins T.W., Sahakian B.J. (2008). Orbitofrontal dysfunction in patients with obsessive-compulsive disorder and their unaffected relatives. Science.

[b0045] Cox J., Witten I.B. (2019). Striatal circuits for reward learning and decision-making. Nat. Rev. Neurosci..

[b0050] Cushman F., Morris A. (2015). Habitual control of goal selection in humans. Proc. Natl. Acad. Sci..

[b0055] de Wit S., Watson P., Harsay H.A., Cohen M.X., van de Vijver I., Ridderinkhof K.R. (2012). Corticostriatal connectivity underlies individual differences in the balance between habitual and goal-directed action control. J. Neurosci..

[b0060] del Casale A., Kotzalidis G.D., Rapinesi C., Serata D., Ambrosi E., Simonetti A., Pompili M., Ferracuti S., Tatarelli R., Girardi P. (2011). Functional neuroimaging in obsessive-compulsive disorder. Neuropsychobiology.

[b0065] Di Martino A., Scheres A., Margulies D.S., Kelly A.M.C., Uddin L.Q., Shehzad Z., Biswal B., Walters J.R., Castellanos F.X., Milham M.P. (2008). Functional connectivity of human striatum: a resting state fMRI study. Cereb. Cortex.

[b0070] Dolan R.J., Dayan P. (2013). Goals and habits in the brain. Neuron.

[b0075] Dong C., Yang Q., Liang J., Seger C.A., Han H., Ning Y., Chen Q.i., Peng ZiWen (2020). Impairment in the goal-directed corticostriatal learning system as a biomarker for obsessive–compulsive disorder. Psychol. Med..

[b0080] Fajnerova I., Gregus D., Francová A., Nosková E., Koprivova J., Stopkova P., Hlinka J., Horacek J. (2020). Functional connectivity changes in obsessive-compulsive disorder correspond to interference control and obsessions severity. Front. Neurol..

[b0085] Fernandez-Egea E., Yulia W., Bernardo M., Robbins T. (2018). Distinct risk factors for obsessive and compulsive symptoms in chronic schizophrenia. Psychol. Med..

[b0090] Figee M., Pattij T., Willuhn I., Luigjes J., van den Brink W., Goudriaan A., Potenza M.N., Robbins T.W., Denys D. (2016). Compulsivity in obsessive–compulsive disorder and addictions. Eur. Neuropsychopharmacol..

[b0095] First, M.B., Spitzer, R.L., Gibbon, M., Williams, J.B.W., 2002. Structured Clinical Interview for DSM-IV-TR Axis I Disorders, Patient Edition (SCID-I/P, 11/2002 revision), for DSMIV.

[b0100] Foa E.B., Huppert J.D., Leiberg S., Langner R., Kichic R., Hajcak G., Salkovskis P.M. (2002). The obsessive-compulsive inventory: development and validation of a short version. Psychol. Assess..

[b0105] Geramita M.A., Yttri E.A., Ahmari S.E. (2020). The two-step task, avoidance, and OCD. J. Neurosci. Res..

[b0110] Gillan C.M., Apergis-Schoute A.M., Morein-Zamir S., Urcelay G.P., Sule A., Fineberg N.A., Sahakian B.J., Robbins T.W. (2015). Functional neuroimaging of avoidance habits in obsessive-compulsive disorder. Am. J. Psychiatry.

[b0115] Gillan C.M., Morein-Zamir S., Kaser M., Fineberg N.A., Sule A., Sahakian B.J., Cardinal R.N., Robbins T.W. (2014). Counterfactual processing of economic action-outcome alternatives in obsessive-compulsive disorder: further evidence of impaired goal-directed behavior. Biol. Psychiatry.

[b0120] Gillan C.M., Morein-Zamir S., Urcelay G.P., Sule A., Voon V., Apergis-Schoute A.M., Fineberg N.A., Sahakian B.J., Robbins T.W. (2014). Enhanced avoidance habits in obsessive-compulsive disorder. Biol. Psychiatry.

[b0125] Gillan C.M., Papmeyer M., Morein-Zamir S., Sahakian B.J., Fineberg N.A., Robbins T.W., de Wit S. (2011). Disruption in the balance between goal-directed behavior and habit learning in obsessive-compulsive disorder. Am. J. Psychiatry.

[b0130] Gillan C.M., Robbins T.W. (2014). Goal-directed learning and obsessive–compulsive disorder. Philos. Trans. R. Soc. B Biol. Sci..

[b0135] Gillan C.M., Robbins T.W., Sahakian B.J., van den Heuvel O.A., van Wingen G. (2016). The role of habit in compulsivity. Eur. Neuropsychopharmacol..

[b0140] Gonçalves Ó.F., Carvalho S., Leite J., Fernandes-Gonçalves A., Carracedo A., Sampaio A. (2016). Cognitive and emotional impairments in obsessive compulsive disorder: evidence from functional brain alterations. Porto Biomed. J..

[b0145] Goodman W.K., Price L.H., Rasmussen S.A., Mazure C., Delgado P., Heninger G.R., Charney D.S. (1989). The yale-brown obsessive compulsive scale. II. Validity. Arch Gen Psychiatry.

[b0150] Gottesman I.I., Gould T.D. (2003). The endophenotype concept in psychiatry: etymology and strategic intentions. Am. J. Psychiatry.

[b0155] Gottesman I.I., Shields J. (1973). Genetic theorizing and schizophrenia. Br. J. Psychiatry.

[b0160] Gu B.-M., Park J.-Y., Kang D.-H., Lee S.J., Yoo S.Y., Jo H.J., Choi C.-H., Lee J.-M., Kwon J.S. (2007). Neural correlates of cognitive inflexibility during task-switching in obsessive-compulsive disorder. Brain.

[b0165] Han J., Kang D.-H., Gu B.-M., Jung W.H., Choi J.-S., Choi C.-H., Jang J., Kwon J.S. (2011). Altered brain activation in ventral frontal-striatal regions following a 16-week pharmacotherapy in unmedicated obsessive-compulsive disorder. J. Korean Med. Sci..

[b0170] Heilbronner S.R., Rodriguez-Romaguera J., Quirk G.J., Groenewegen H.J., Haber S.N. (2016). Circuit-based corticostriatal homologies between rat and primate. Biol. Psychiatry.

[b0175] Howard J.D., Kahnt T. (2017). Identity-specific reward representations in orbitofrontal cortex are modulated by selective devaluation. The Journal of Neuroscience.

[b0180] Hu X., Du M., Chen L., Li L., Zhou M., Zhang L., Liu Q.i., Lu L.u., Mreedha K., Huang X., Gong Q. (2017). Meta-analytic investigations of common and distinct grey matter alterations in youths and adults with obsessive-compulsive disorder. Neurosci. Biobehav. Rev..

[b0185] Mayr U., Keele S.W. (2000). Changing internal constraints on action: the role of backward inhibition. J. Exp. Psychol. Gen..

[b0190] Medaglia J.D., Huang W., Karuza E.A., Kelkar A., Thompson-Schill S.L., Ribeiro A., Bassett D.S. (2018). Functional alignment with anatomical networks is associated with cognitive flexibility. Nat. Hum. Behav..

[b0195] Menzies L., Achard S., Chamberlain S.R., Fineberg N., Chen C.-H., del Campo N., Sahakian B.J., Robbins T.W., Bullmore E. (2007). Neurocognitive endophenotypes of obsessive-compulsive disorder. Brain.

[b0200] Middleton F. (2000). Basal ganglia and cerebellar loops: motor and cognitive circuits. Brain Res. Rev..

[b0205] Milad M.R., Rauch S.L. (2012). Obsessive-compulsive disorder: beyond segregated cortico-striatal pathways. Trends Cognit. Sci..

[b0210] Monsell S. (2003). Task switching. Trends Cognit. Sci..

[b0215] Moody, T., Morfini, F., Cheng, G., Sheen, C., Tadayonnejad, R., Reggente, N., O’Neill, J., Feusner, J., 2017. Mechanisms of cognitive-behavioral therapy for obsessive-compulsive disorder involve robust and extensive increases in brain network connectivity. Translational Psychiatry 7, e1230. https://doi.org/10.1038/tp.2017.192. 10.1038/tp.2017.192 PMC5639240 28872637

[b0220] Morelli E., Moore H., Rebello T.J., Gray N., Steele K., Esposito E., Gingrich J.A., Ansorge M.S. (2011). Chronic 5-HT transporter blockade reduces DA signaling to elicit basal ganglia dysfunction. J. Neurosci..

[b0225] Nagarajan N., Jones B.W., West P.J., Marc R.E., Capecchi M.R. (2018). Corticostriatal circuit defects in Hoxb8 mutant mice. Mol. Psychiatry.

[b0230] Nestadt G., Grados M., Samuels J.F. (2010). Genetics of obsessive-compulsive disorder. Psychiatr. Clin. North Am..

[b0235] O’Doherty J.P., Cockburn J., Pauli W.M. (2017). Learning, reward, and decision making. Annu. Rev. Psychol..

[b0240] Page L., Rubia K., Deeley Q., Daly E., Toal F., Mataix-Cols D., Giampietro V., Schmitz N., Murphy D. (2009). A functional magnetic resonance imaging study of inhibitory control in obsessive–compulsive disorder. Psychiatry Res.

[b0245] Pauls D.L. (2008). The genetics of obsessive compulsive disorder: a review of the evidence. Am. J. Med. Genet. Part C Semin. Med. Genet..

[b0250] Pauls D.L., Abramovitch A., Rauch S.L., Geller D.A. (2014). Obsessive–compulsive disorder: an integrative genetic and neurobiological perspective. Nat. Rev. Neurosci..

[b0255] Peng Z., Li G., Shi F., Shi C., Yang Q., Chan R.C.K., Shen D. (2015). Cortical asymmetries in unaffected siblings of patients with obsessive–compulsive disorder. Psychiatry Res. Neuroimag..

[b0260] Peng Z., Yang W., Miao G., Jing J., Chan R.C. (2011). The Chinese version of the obsessive-compulsive inventory-revised scale: Replication and extension to non-clinical and clinical individuals with OCD symptoms. BMC Psychiatry.

[b0265] Pinhal C.M., van den Boom B.J.G., Santana-Kragelund F., Fellinger L., Bech P., Hamelink R., Feng G., Willuhn I., Feenstra M.G.P., Denys D. (2018). Differential effects of deep brain stimulation of the internal capsule and the striatum on excessive grooming in Sapap3 mutant mice. Biol. Psychiatry.

[b0270] Remijnse, P., Heuvel, O., Nielen, M., Vriend, C., Hendriks, G.-J., Hoogendijk, W., Uylings, H., Veltman, D., 2013. Cognitive Inflexibility in Obsessive-Compulsive Disorder and Major Depression Is Associated with Distinct Neural Correlates. PLoS One 8, e59600. https://doi.org/10.1371/journal.pone.0059600. 10.1371/journal.pone.0059600 PMC3634812 23637737

[b0275] Robbins T.W., Vaghi M.M., Banca P. (2019). Obsessive-compulsive disorder: puzzles and prospects. Neuron.

[b0280] Rotge J.-Y., Guehl D., Dilharreguy B., Cuny E., Tignol J., Bioulac B., Allard M., Burbaud P., Aouizerate B. (2008). Provocation of obsessive-compulsive symptoms: a quantitative voxel-based meta-analsysis of functional neuroimaging studies. J. Psychiatry Neurosci..

[b0285] Seger C.A. (2018). Corticostriatal foundations of habits. Curr. Opin. Behav. Sci..

[b0290] Sha Z., Edmiston E.K., Versace A., Fournier J.C., Graur S., Greenberg T., Lima Santos J.P., Chase H.W., Stiffler R.S., Bonar L., Hudak R., Yendiki A., Greenberg B.D., Rasmussen S., Liu H., Quirk G., Haber S., Phillips M.L. (2020). Functional disruption of cerebello-thalamo-cortical networks in obsessive-compulsive disorder. Biol. Psychiatry Cogn. Neurosci. Neuroimaging.

[b0295] Simmler L.D., Ozawa T. (2019). Neural circuits in goal-directed and habitual behavior: implications for circuit dysfunction in obsessive-compulsive disorder. Neurochem. Int..

[b0300] Spielberger C.D. (1989).

[b0305] Spitzer M.B., Robert L., Gibbon M., Gibbons W., Janet B.W., Gibbon Miriam R.L., Williams J.B.W. (2002).

[b0310] Thorsen A.L., Hagland P., Radua J., Mataix-Cols D., Kvale G., Hansen B., van den Heuvel O.A. (2018). Emotional processing in obsessive-compulsive disorder: a systematic review and meta-analysis of 25 functional neuroimaging studies. Biol. Psychiatry Cognit. Neurosci. Neuroimag..

[b0315] Thorsen A.L., van den Heuvel O.A., Hansen B., Kvale G. (2015). Neuroimaging of psychotherapy for obsessive-compulsive disorder: a systematic review. Psychiatry Res. Neuroimag..

[b0320] van der Plasse G., La Fors S.S.B.M., Meerkerk D.T.J., Joosten R.N.J.M.A., Uylings H.B.M., Feenstra M.G.P. (2007). Medial prefrontal serotonin in the rat is involved in goal-directed behaviour when affect guides decision making. Psychopharmacology.

[b0325] Viviani R. (2014). Neural correlates of emotion regulation in the ventral prefrontal cortex and the encoding of subjective value and economic utility. Front. Psychiatry.

[b0330] Wolff N., Buse J., Tost J., Roessner V., Beste C. (2017). Modulations of cognitive flexibility in obsessive compulsive disorder reflect dysfunctions of perceptual categorization. J. Child Psychol. Psychiatry.

[b0335] Woolley J., Heyman I., Brammer M., Frampton I., McGuire P.K., Rubia K. (2008). Brain activation in paediatric obsessive compulsive disorder during tasks of inhibitory control. Br. J. Psychiatry.

[b0340] Xu T.-T., Zhao Q., Wang P., Fan Q., Chen J., Zhang H., Yang Z., Stein D., Wang Z. (2018). Altered resting-state cerebellar-cerebral functional connectivity in obsessive-compulsive disorder. Psychol. Med..

[b0345] Yin H.H., Knowlton B.J. (2006). The role of the basal ganglia in habit formation. Nat. Rev. Neurosci..

