# Supplementary material for: Imbalance between the caudate and putamen connectivity in obsessive–compulsive disorder
Source: Neuroimage Clin. 2022 Jun 14;35:103083. doi: 10.1016/j.nicl.2022.103083 (PMC9213242; doi:10.1016/j.nicl.2022.103083)
Supplement: Supplementary data 1 [file mmc1.docx]

**Supplementary Material**

**Table S1.** Treatment details of OCD patients.

| **Treatment** | **Number of case** | **Average dosage (mg)** | |
| --- | --- | --- | --- |
| citalopram | 4 | | 40 |
| clomipramine | 2 | | 100 |
| escitalopram | 4 | | 20 |
| fluoxetine | 1 | | 40 |
| mirtazapine | 2 | | 30 |
| paroxetine | 3 | | 40 |
| sertraline | 8 | | 150 |
| paroxetine + sodium valproate | 3 | | 40 + 500 |
| paroxetine + quetiapine | 4 | | 40 + 200 |


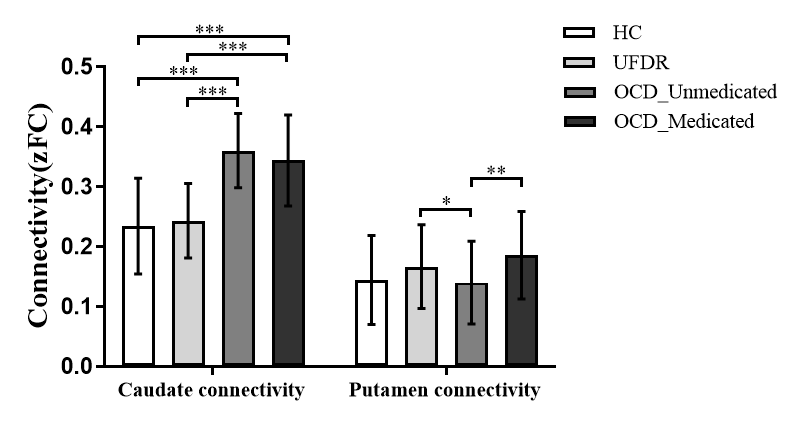


**Fig. S1. The contribution of medicine between groups.**

In order to assess the effect bringing by the medicine, we conducted group comparison between groups based on the mean strength of caudate connectivity and putamen connectivity (nuisance variables were taken into consideration). The results showed that there were no significant differences between medicated patients and unmedicated patients in the caudate connectivity (*p=*0.327). On the caudate connectivity, the connectivity strengths in medicated and unmedicated patients were all significantly higher than that in HC and UFDR group, while the differences between HC and UFDR group were not obvious (*p=*0.203). On the putamen connectivity, the connectivity strengths in unmedicated patients were significantly lower than that in unmedicated patients and UFDR group, while the differences between HC and the other groups were not obvious (*p*>0.05). **p*<0.05, ***p*<0.01, ****p*<0.001.

**The task-switching paradigm**

The task-switching paradigm (programmed in E-prime 2.0) was conducted outside the MRI scanner. In this paradigm, there were two types of picture (diamond picture, square picture) as the cue, four kinds of icons (green face, blue face, green house and blue house) as the stimuli, and the subjects were required to press keys for discriminating these stimuli according to color (green, blue) or shape (house, face). During each trial, the subjects were cued explicitly (with a diamond or square cue) as to which task would be performed during the presentation of next stimulus. When the presentation of diamond cue, the subjects were required to press keys to discriminate the color (green or blue); when the square cue was presented, they were required to press keys to discriminate the shape (house or face). Prior to the test trial, each subject learned and practiced stimulus-response mapping about 5 minutes. Stimulus-response mapping was counterbalanced among the subjects. Half of the subjects, they pressed the same key when presented green icons during the color condition consistent with house icons during the shape condition; for the other half, green icons corresponded to the same key as face icons. All of the 96 trials were divided equally into two task conditions. In task-switching conditions, the subjects attended to a different dimension from the previous one and should change the stimulus-response set, like as a diamond cue followed by a square cue. In task-repeat conditions, they didn’t need to change the stimulus-response set. At last, according to the performances of these subjects, the reaction time (RT) and response accuracy (AC) during the task-switching (switch-RT and switch-AC) and task-repeat condition (repeat-RT and repeat-AC) were calculated as the indicators. The detailed experimental materials and procedure displayed in Fig. S2a and Fig. S2b.


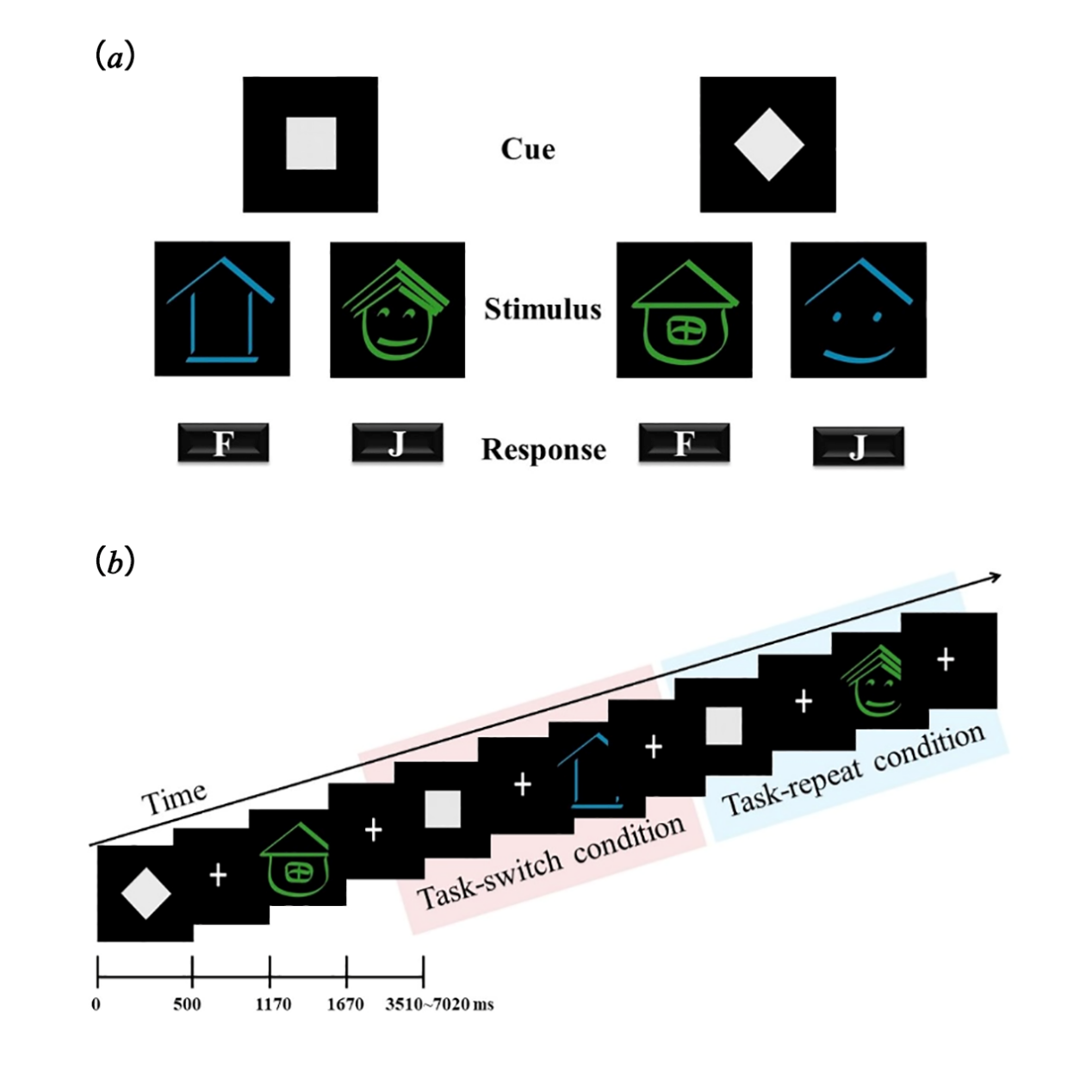


**Fig. S2. (a)** Materials of the task-switching paradigm. **(b)** The time flow of the trials.


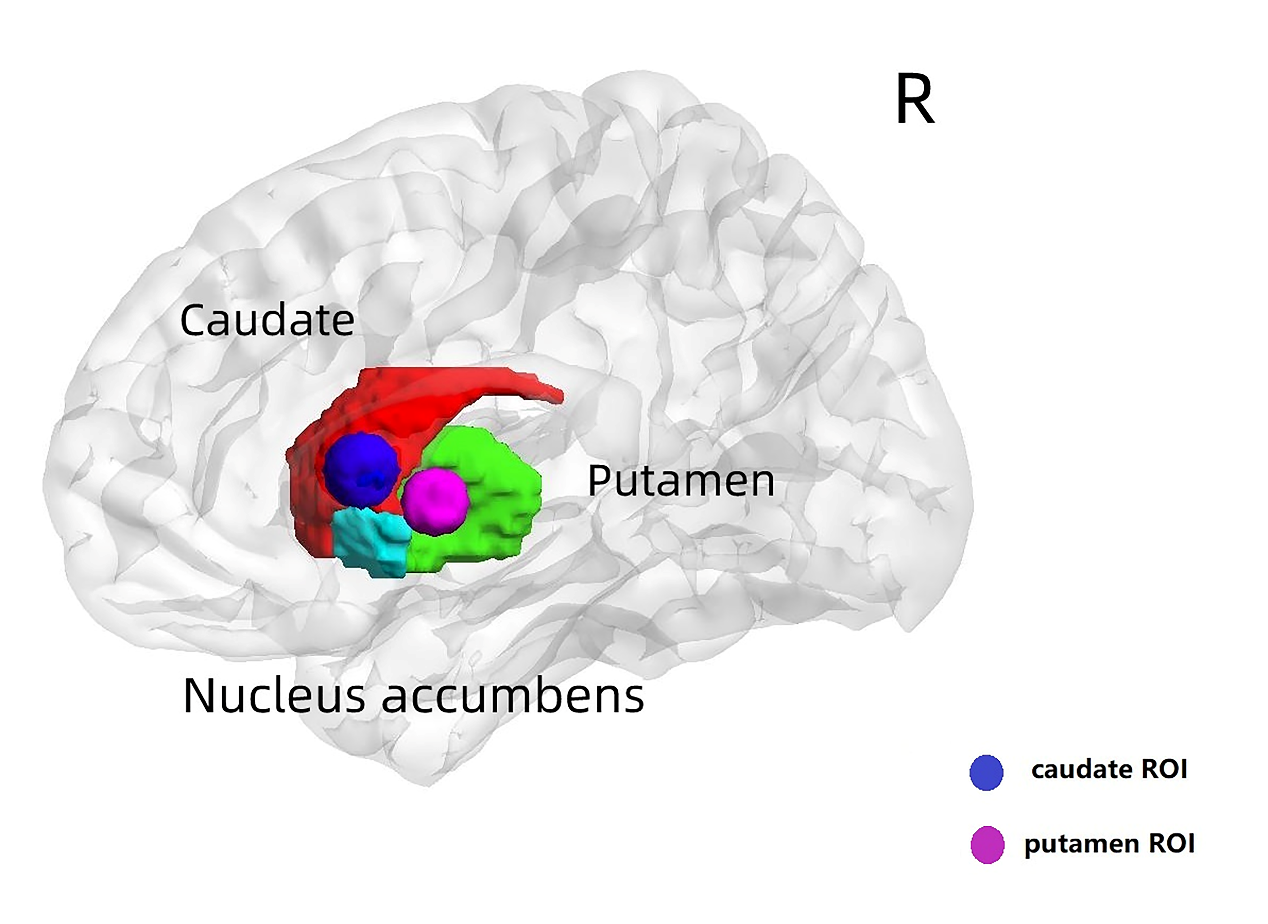


**Fig. S3. The location of two spherical ROI.**

We draw the above plot to show the location of two spherical ROI for showing no overlaps between them. The blue ball represents the caudate spherical ROI and the pink ball represents the putamen spherical ROI.

**Table S2.** The mean RT (ms) and mean ER (%) in switching task.

| **Group** | **Reaction** | **Task repeat**  **M(SD)** | **Task switch**  **M(SD)** | **Switching cost**  **M(SD)** |
| --- | --- | --- | --- | --- |
| OCD | RT | 661.69(230.165) | 1643.38(460.33) | 981.69(230.165) |
|  | ER | 22.14(17.59) | 24.38(18.29) | 22.14(17.59) |

OCD, obsessive-compulsive disorder; RT, reaction time; ER, error rate; M, mean; SD: standard deviation.
